# Association of miRNA − 320 expression level and its target gene endothelin-1 with the susceptibility and clinical features of polycystic ovary syndrome

**DOI:** 10.1186/s13048-019-0513-5

**Published:** 2019-05-07

**Authors:** Nearmeen M. Rashad, Marwa Abdel-Monem Ateya, Yasser S. Saraya, Walid Mohamed Elnagar, Khaled Fathy Helal, Mohamed El-Bakry Lashin, Amr Ahmed Abdelrhman, Ayman E. Alil, Mohammed S. Yousef

**Affiliations:** 1 Internal Medicine Department, Faculty of Medicine, Zagazig University, Zagazig, Egypt; 2 Clinical Pathology Department, Faculty of Medicine, Zagazig University, Zagazig, Egypt; 3 Obstetrics and Gynecology Department, Faculty of Medicine, Zagazig University, Zagazig, Egypt

**Keywords:** miR-320, PCOS, Gene expression, ET-1, Insulin resistance

## Abstract

**Background:**

Polycystic ovary syndrome (PCOS) is a common reproductive endocrine disorder characterized by obesity, hyperandrogenism, and insulin resistance (IR). MicroRNAs (miRNAs) are small noncoding RNA associated with ovarian follicle development and female fertility. *The objective* of this study was to investigate the role of miRNA- 320 and its target gene endothelin-1 (ET-1) as a noninvasive biomarker of PCOS and to evaluate its possible relationship with IR as well as clinic-morphological features of PCOS.

**Methods:**

Case-control study enrolled 60 patients with PCOS and 40 control group. We subdivided our PCOS women according to homeostasis model assessments of insulin resistance (HOMA-IR) to PCOS women with and without IR.ET-1 levels were measured by ELISA. We estimated the serum expression level of miRNA- 320 by real-time polymerase chain reaction.

**Results:**

Our results revealed that serum miR-320 expression level was lower in PCOS patients compared to controls, in particular, PCOS women with IR. Moreover, it was negatively correlated to its target gene; ET-I as well as fasting serum insulin (FSI), HOMA-IR, PCOS phenotype; hirsutism score, ovarian volume and antral follicle count (AFC). In the PCOS group, linear regression analysis revealed that only hirsutism and HOMA-IR was the main predictor of expression levels of miRNA − 320 among other clinical and laboratory biomarkers of PCOS. The sensitivity and specificity of serum miR-320 expression levels in diagnosis PCOS was 80, and 97.5% respectively.

**Conclusion:**

The Expression serum levels of miR-320 were lower in PCOS compared to control and it could be a noninvasive diagnostic biomarker of PCOS.

## Introduction

Compelling evidence suggests that polycystic ovary syndrome (PCOS) is the commonest reproductive endocrine disease of women as affecting 5–10% of women in reproductive age [1]. Several lines of evidence indicate that PCOS patients suffer from metabolic disorders, which is manifested by obesity, type 2 diabetes mellitus(T2DM) and insulin resistance (IR), in this respect, about 50–70% of PCOS patients have various degree of IR [2]. A growing body of evidence has corroborated the association between IR and hyperandrogenism among PCOS women [3].

MicroRNAs (miRNAs) are small, non-coding RNAs (19–23 nucleotides) that inhibit translation and/or direct mRNA degradation, in addition they have an autocrine and endocrine regulatory function of gene expression and involved in the pathogenesis of complex diseases including obesity [4], T2DM [5], and PCOS [6, 7]. MiR-320 has been shown to be involved in growth, proliferation, and the cell cycle by targeting different genes in different cell lines [8]. However, the functions and mechanisms of miR-320 in PCOS remain unclear. MiR-320 is supposed to have widespread biological effects as it regulates multiple important molecules in particular endothelin (ET-1). Mounting evidence indicates that MiR-320 target gene; ET is a bioactive peptide produced by endothelial cells that can promote cell mitosis, participate in tumor growth and induce mitosis in tumor growth [9]. Three types of ET have been identified, ET-1, ET-2, and ET-3, of which ET-1 is the most potent biomolecule [10].

Emerging evidence demonstrated that genetic factors in PCOS pathogenesis, of particular interest the genes that are involved in the etiology of the syndrome have not been fully investigated until now, as well as the environmental contribution in their expression. The aim of the current study was to investigate the role of miRNA- 320 as a noninvasive biomarker of PCOS and to evaluate its possible relationship with IR as well as clinic-morphological features of PCOS.

## Materials and methods

This case-control study included 100 unrelated subjects. Sixty women with PCO recruited from Outpatient Clinics of the Endocrinology Unit of Internal Medicine and Obstetrics and Gynecology Departments, Faculty of Medicine, Zagazig University, Egypt and 40 healthy women matched to PCOS women as regard age, and ethnic origin. Patients were divided into two groups; PCOS without IR [(homeostatic model assessment-IR) HOMA –IR <2.11), *n* = 35] and PCOS with IR [(HOMA -IR > 2.11), *n* = 25]. The diagnosis of PCOS was based on the 2004 revised Rotterdam criteria [11]. All women underwent a menstrual history and a thorough clinical examination. All patients were subjected to thorough history taking, full clinical assessment and anthropometric measures of obesity. Ovarian volume and antral follicular count were evaluated by transvaginal ultrasound (TVS). The exclusion criteria for all women included a history of hyperandrogenic states (such as non-classical congenital adrenal hyperplasia, androgen-secreting tumors, Cushing’s syndrome, 21-hydroxylase deficiency, or hyperprolactinemia), hypertension, liver, kidney, or thyroid diseases.

### Ethics approval and consent to participate

Written informed consent was taken from all of the participants. The ethical committee of Faculties of Medicine, Zagazig University approved this study.

#### The sampling of blood and laboratory assessments

Blood samples were drawn from all subjects during the early follicular phase of the menstrual cycles after an overnight fast. Fasting plasma glucose (FPG), total cholesterol (TC), high-density lipoprotein (HDL) cholesterol, triglycerides level and low-density lipoprotein (LDL) cholesterol level were determined using automated analyzer (Roche Cobas 8000-c702, Roche Diagnostics, Germany).

Serum ET-1 levels were estimated using a quantitative sandwich ELISA method according to manufacturer’s instructions (R& D Minneapolis, MN, USA) ELISA kit. We measured, fasting serum insulin (FSI), follicle stimulating hormone (FSH), luteinizing hormone (LH), total testosterone, we calculated insulin resistance (IR) with the HOMA-IR index, which is defined as the FSI value (lU/mL) × FPG value (mg/dl)/405. The β-cell function was calculated using HOMA-β as follows: {20*[FSI (μU/mL)]/[FPG (mmol/L) - 3.5]}. the laboratory assessments were done at the Clinical Pathology Department in Faculty of Medicine, Zagazig University, Egypt.

#### MicroRNA extraction from sera

miRNAs were extracted from serum samples of the patients and control subjects using the miRNeasy mini kit (Qiagen, Germany); according to the manufacturer’s instructions. The concentrations and purity of the miRNAs were determined using Qubit® 3 Fluorometer (Thermo Fisher Scientific Inc., USA).

#### Reverse transcription of mi RNA to complementary DNA

Reverse transcription reaction was performed using the miScript II RT Kit (Qiagen, Germany) according to the manufacturer’s instruction.

#### Real-time polymerase chain reaction

Amplification was performed using the Stratagene Mx3005P platform (Agilent Technologies, USA) and QuantiFast SYBR Green PCR Kit, (Qiagen, Germany). Primers design was forward, 5′-ACACTCCAGCTGGGAAAAGCTGGGTTGAGA-3′; reverse, 5′- TGGTGTCGTGGAGTCG-3. The reaction mixture was prepared according to manufacture recommendation with total volume of 20 μl: 10 μl 2x QuantiFast SYBR Green PCR Master Mix, 2 μl 10x forward primer, 2 μl 10x reverse primer, 4 μl RNase free water and 2 μl of c DNA using the following cyclic condition: initial activation 15 min at 95 °C then 40 cycles of 15 s at 94 °C,30 s at 55 °C and 30 s at 70 °C. Expression values were obtained as the relative gene (Δ CT = Target gene CT –Reference gene CT).

#### Statistical analysis

Statistical analyses were performed using the Statistical Package for the Social Sciences (version 21.0; SPSS, Chicago, IL, USA). The comparison between two groups with parametric variables was done using independent sample t-test (t) and nonparametric variables using the Mann–Whitney test (z). Correlation analysis was performed using the Pearson correlation method. Linear regression analyses were done to test the influence of the main independent variables against serum miRNA − 320 expression levels in PCOS patients. To test the independent variables associated with PCOS logistic regression analyses were performed. Receiver operating characteristic (ROC) analysis was performed to assess the diagnostic power of serum miRNA − 320 expression levels. We considered P to be significant at < 0.05 with a 95% confidence interval (CI).

## Results

Among studied patients the mean age of the control group was 32.38 ± 7.68 year. While in the PCOS groups the mean age was 31.95 ± 7.42. Control and PCOS patients were matched for age and gender. To assess the association of miRNA − 320 with IR we subclassified PCOS group to PCOS without IR HOMA –IR <2.11), and PCOS with IR (HOMA -IR > 2.11. Comparison of the clinical and laboratory characteristics between patients and control are presented in Table 1.

**Table 1:** Clinical, anthropometric and laboratory characteristics of studied groups

	Control group (mean ± SD) (*n* = 40)	PCO patients (mean ± SD) (*n* = 60)	*P*
Systolic blood pressure (mm Hg)	125.4 ± 7.16	130.6 ± 7.3	<0.001*
Diastolic blood pressure (mm Hg)	85.6 ± 3.96	87.5 ± 4.9	<0.001*
Hirsutism score	5.38 ± 0.594	7.41 ± 3.02	<0.001*
Body mass index (kg/m2)	24.9 ± 2.48	33.2 ± 5.731	<0.001*
Waist/hip ratio	0.98 ± 0.191	1.26 ± 0.28	<0.001*
Ovarian volume	3.58 ± 0.612	7.35 ± 2.744	<0.001*
AFC	2.58 ± 0.612	7.35 ± 2.744	<0.001*
Total cholesterol (mg/dL)	167.5 ± 19.26	185.0 ± 124.7	<0.001*
Triglycerides (mg/dL)	144.55 ± 20.4	192.2 ± 42.05	<0.001*
LDL cholesterol (mg/dL)	106.24 ± 4.24	125.8 ± 18.11	<0.001*
HDL cholesterol (mg/dL)	57.5 ± 7.33	40.3 ± 9.42	<0.001*
Fasting plasma glucose (mg/dL)	83.9 ± 8.40	95.3 ± 15.61	<0.001*
HbA1c (%)	5.5 ± 0.596	6.03 ± 0.42	<0.001*
Fasting serum insulin (lU/mL)	6.8 ± 1.44	17.5 ± 10.41	<0.001^*^
HOMA-IR	1.42 ± 0.322	3.7 ± 2.32	<0.001*
HOMA-β	166.9 ± 76.9	127.7 ± 81.84	0.016
FSH (mIU/mL)	4.8 ± 0.972	5.5 ± 1.538	<0.001*
LH (mIU/mL)	5.52 ± 1.21	6.97 ± 1.53	<0.001*
LH/FSH	1.2 ± 0.43	13 ± 0.39	0.145
DHEA-S (mg/mL)	0.99 ± 0.31	1.3 ± 0.77	<0.001*
Androstenedione (ng/mL)	1.17 ± 0.34	1.7 ± 0.5	0.015
Total testosterone (ng/mL)	0.50 ± 0.145	0.7 ± 0.32	<0.001*

*FSI* fasting serum insulin, *FPG* fasting plasma glucose, *AFC* antral follicle cells, *HOMA-IR* homeostasis model assessments of insulin resistance, *DHEA* dehydroepiandrosterone, *P < 0.05 when compared with control group

### Clinical and laboratory characteristics of studied group

As expected, our study revealed that there were significantly higher values of PCOS phenotype features in both PCOS groups especially IR group as compared to control group. In addition, IR group had significantly higher values of hyperlipidemia and hyperglycemia compared to control group as shown in Table 2, *p* <0.001. Among the PCOS group, IR patients had significantly higher values of body composition parameters; body mass index (BMI) and waist/hip ratio. Also, systolic blood pressure as well as, TC, LDL, FSI, HbA1c, and HOMA-IR compared to PCOS women without IR. In addition, PCOS phenotypes; LH, DHEA-S androstenedione, and total testosterone, were significantly high in IR patients compared to PCOS women without IR. On the contrary, we detected significant lower HDL and HOMA-β levels in the IR group compared to PCOS women without IR, Table 2 *P* <0.001*.

**Table 2:** Clinical, anthropometric and laboratory characteristics of studied groups

	Control group(mean ± SD) (*n* = 40)	Non-IR group (mean ± SD) (*n* = 25)	IR group (mean ± SD) (*n* = 35)	P 1	P2	P3
SBP (mm Hg)	125.4 ± 7.16	126.96 ± 8.33	133.2 ± 5.19	0.37	<0.001*	<0.001*
DBP (mm Hg)	85.6 ± 3.96	86.28 ± 4.39	88.37 ± 5.1	0.555	0.009	0.104
Hirsutism score	5.38 ± 0.594	7.34 ± 4.49	7.45 ± 1.2	<0.001*	<0.001*	0.893
Body mass index (kg/m2)	24.9 ± 2.48	29.1 ± 5.96	36.18 ± 3.2	<0.001*	<0.001*	<0.001*
Waist/hip ratio	0.98 ± 0.191	1.11 ± 0.28	1.36 ± 0.2	<0.05*	<0.001*	<0.001*
Ovarian volume	3.58 ± 0.612	7.2 ± 4.56	7.85 ± 1.437	<0.001*	<0.001*	0.467
AFC	2.58 ± 0.612	7.3 ± 4.02	7.37 ± 1.285	<0.001*	<0.001*	0.952
Total cholesterol (mg/dL)	167.5 ± 19.26	167.1 ± 25.15	197.8 ± 14.4	0.916	<0.001*	<0.001*
Triglycerides (mg/dL)	144.55 ± 20.4	193.2 ± 58.2	191.6 ± 25.9	<0.001*	<0.001*	0.883
LDL cholesterol (mg/dL)	106.24 ± 4.24	114.3 ± 17.4	134.06 ± 13.6	0.01	<0.001*	<0.001*
HDL cholesterol (mg/dL)	57.5 ± 7.33	45.8 ± 9.27	36.4 ± 7.48	<0.001*	<0.001*	<0.001*
Fasting plasma glucose (mg/dL)	83.9 ± 8.40	93.7 ± 16.19	96.4 ± 15.3	<0.001*	<0.001*	0.516
HbA1c (%)	5.5 ± 0.596	5.8 ± 0.171	6.18 ± 0.49	0.01	<0.001*	<0.001*
Fasting serum insulin (lU/mL)	6.8 ± 1.44	7.2 ± 1.29	24.97 ± 7.17	0.755	<0.001*	<0.001*
HOMA-IR	1.42 ± 0.322	1.65 ± 0.3	5.25 ± 1.92	0.448	<0.001*	<0.001*
HOMA-β	166.9 ± 76.9	149.3 ± 18.3	86.2 ± 22.5	0.384	<0.001*	<0.001*
FSH (mIU/mL)	4.8 ± 0.972	5.14 ± 1.27	5.85 ± 1.65	0.313	<0.001*	0.078
LH (mIU/mL)	5.52 ± 1.21	6.1 ± 1.2	7.59 ± 1.4	0.081	<0.001*	<0.001*
LH/FSH	1.2 ± 0.43	7.59 ± 1.4	1.3 ± 0.4	0.778	<0.001*	0.121
DHEA-S (mg/mL)	0.99 ± 0.31	0.99 ± 0.34	1.5 ± 0.91	0.967	<0.001*	<0.001*
Androstenedione (ng/mL)	1.17 ± 0.34	1.35 ± 0.48	2.07 ± 0.46	0.103	<0.001*	<0.001*
Total testosterone (ng/mL)	0.50 ± 0.145	0.62 ± 0.28	0.9 ± 0.3	0.062	<0.001*	<0.001*

IR; *FSI* fasting serum insulin, *FPG* fasting plasma glucose, *AFC* antral follicle cells, *HOMA-IR* homeostasis model assessments of insulin resistance, *DHEA* dehydroepiandrosterone

^1^Significant *P* values (P < 0.05) when compared non-IR group with control group

^2^Significant *P* values (*P* < 0.05) when compared IR group with control group ^3^Significant P values (*P* < 0.05) when compared non-IR group with IR group

### Comparison of serum miRNA − 320 expression and serum ET-1(pg/ml) in studied groups

**Fig. 1:**
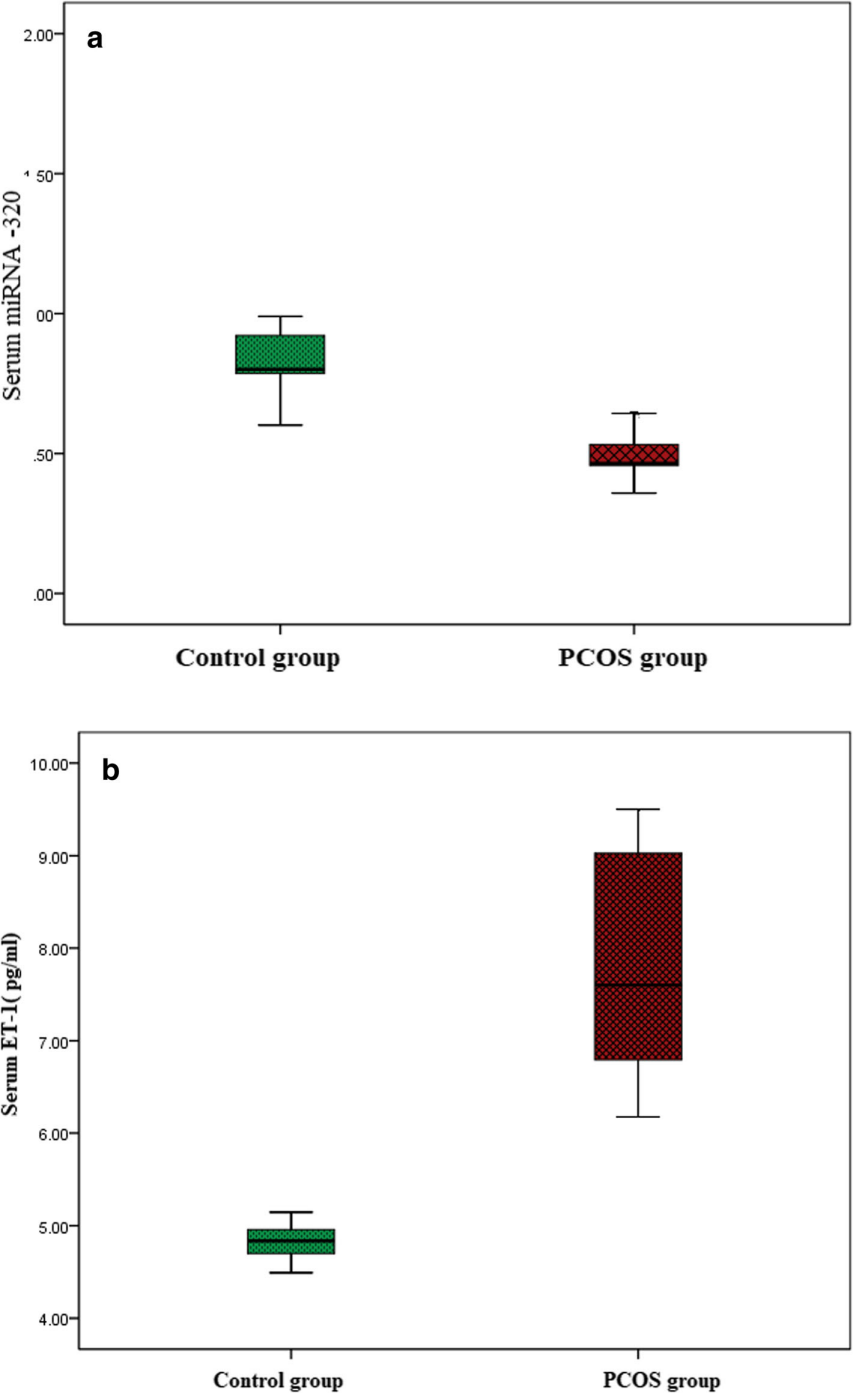
**a** Comparison of serum miRNA − 320 expressions levels in studied groups. **b** Comparison of serum ET-1(pg/ml) in studied groups

Regarding expression levels of miRNA -320, PCOS patients (0.91 ± 0.331) had significantly lower levels compared to the control group (0.49 ± 0.08) )Fig. 1a). Among the PCOS group, IR patients (0.456 ± 0.034) had significantly lower expression levels of miRNA − 320 compared to PCOS women without IR (0.558 ± 0.084) (Fig. 1b), *P* <0.001*.

**Fig. 2:**
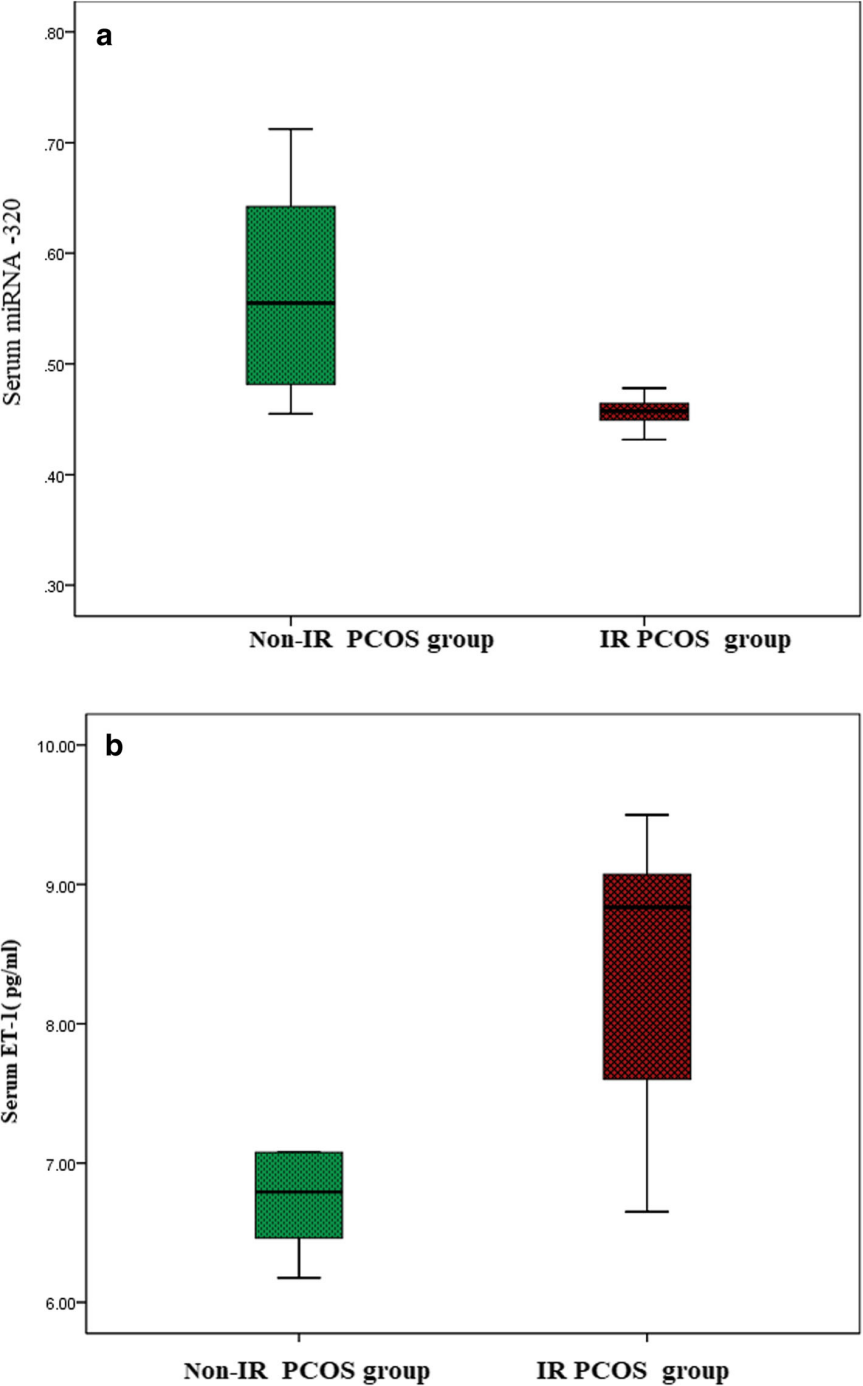
**a** Comparison of serum miRNA − 320 expression level in PCOS women. **b** Comparison of serum ET-1in PCOS women

Regarding serum levels of ET-1, PCOS patients (7.86 ± 1.133) had significantly higher levels compared to the control group (4.82 ± 0.193) (Fig. 2a). Among the PCOS group, IR patients (8.27 ± 0.876) had significantly higher serum levels of ET-1compared to PCOS women without IR (7.14 ± 0.876) (Fig. 2b), *P* <0.001*.

#### Correlation between expression levels of miRNA − 320 with clinical and biochemical parameters of PCOS patients

Serum miRNA − 320 expression levels were significantly negatively correlated with FSI and HOMA-IR, as well as PCOS phenotype; hirsutism score, ovarian volume AFC, and TG. Even more interestingly, miRNA − 320 expression levels were significantly negatively correlated ET-1 with *P* <0.001*, (Table 3).

**Table 3:** Pearson correlation of serum miRNA − 320 expression levels with clinical, anthropometric as well as biochemical characteristics in studied subjects

Characteristics	Serum miRNA − 320 expression levels	
	r	*p*
Hirsutism score	−0.469	<0.001*
Body mass index (kg/m2)	−0.183	0.162
Waist/hip ratio	−0.109	0.406
Ovarian volume	−0.484	<0.001*
AFC	−0.475	<0.001*
Total cholesterol (mg/dL)	− 0.189	0.147
Triglycerides (mg/dL)	−0.375	<0.001*
LDL cholesterol (mg/dL)	−0.061	0.643
HDL cholesterol (mg/dL)	0.226	0.082
FPG (mg/dL)	−0.020	0.882
FSI (lU/mL)	−0.515	<0.001*
HOMA-IR	−0.501	<0.001*
HbA1c (%)	−0.167	0.204
HOMA-β	0.020	0.020
Total testosterone (ng/mL)	0.098	0.456
FSH (mIU/mL)	−0.115	0.383
LH (mIU/mL)	−0.271	0.036
DHEA-S (mg/mL)	−0.190	0.145
Androstenedione (ng/mL)	−0.207	0.112

*FSI* Fasting serum insulin, *FPG* Fasting plasma glucose, *AFC* antral follicle cells, *HOMA-IR* homeostasis model assessments of insulin resistance, *DHEA-S* dehydroepiandrosteron sulfate,*Statistically significant (*P* < 0.05)

#### Linear regression analysis with expression levels of miRNA − 320 as the dependent variable in PCOS groups

In the PCOS group, linear regression analysis revealed that only hirsutism and HOMA-IR was the main predictor of expression levels of miRNA − 320 among other clinical and laboratory biomarkers of PCOS (Table 4).

**Table 4:** linear regression analyses in PCOS women to test the influence of the main independent variables against serum miRNA −320 expression levels (dependent variable) in PCOS women

Model		Unstandardized Coefficients		Standardized Coefficients	t	P value	95% C.I.	
		B	SE	Beta			Lower Bound	Upper Bound
1	Constant	0.497	0.052		9.646	<0.001*	0.394	0.600
	BMI	−0.002	0.002	−0.181	−1.394	0.169	−0.006	0.001
	Hirsutism	0.009	0.004	0.343	2.126	<0.05*	0.000	0.017
	TG	.000	0.000	0.112	0.925	0.359	0.000	0.001
	AFC	0.006	0.005	0.217	1.251	0.216	0–.004	0.016
	DEHA-S	−0.013	0.010	−0.127	−1.314	0.194	−0.032	0.007
	HOMA-IR	−0.013	0.004	−0.397	−3.479	<0.001*	−0.021	−0.006

*Statistically significant (*P* < 0.05)

#### Logistic regression analysis evaluating the main independent variables associated with PCOS

Among the studied parameters, serum miRNA -320 expression levels were independently associated with PCOS, *P* < 0.001* (Table 5).

**Table 5:** Logistic regression analysis evaluating the main independent variables associated with PCOS

		B	S.E.	Wald	*P* value	odds	95% C.I.	
							Lower	Upper
Step 1	MiRNA −320	−12.252	3.326	13.572	<0.001*	0.000	0.000	0.003
	BMI	0.210	0.112	3.552	0.059	1.234	0.992	1.536
	LH	0.350	0.326	1.153	0.283	1.419	0.749	2.688
	Hirsutism	0.097	0.211	0.214	0.644	1.102	0.730	1.665
	Constant	−1.146	2.834	0.164	0.686	0.318		

*Statistically significant (*P* < 0.05)

#### The accuracy of serum miRNA − 320 for diagnosis of PCOS by ROC analysis

**Fig. 3:**
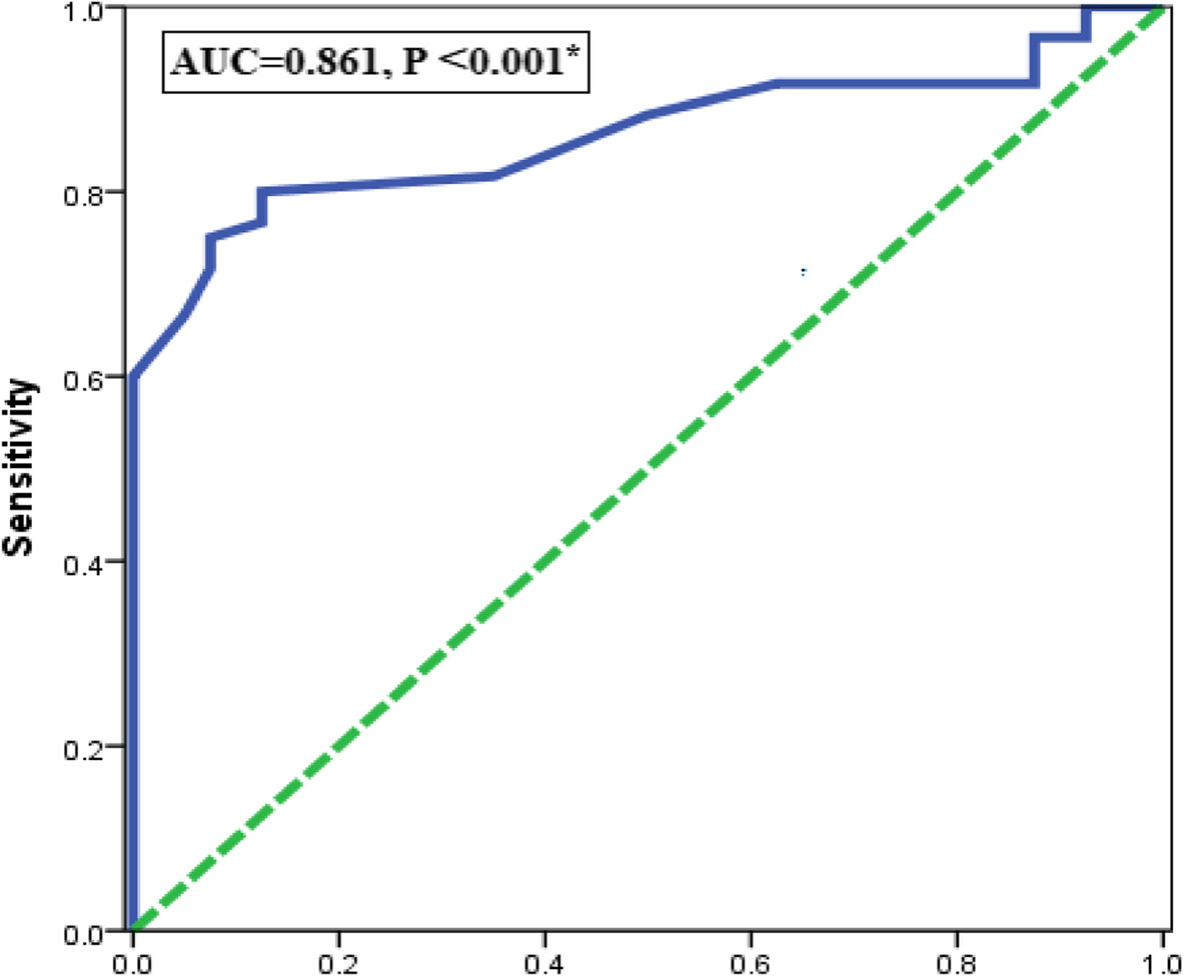
Receiver operating characteristic (ROC) curve for serum miRNA − 320 expression level for prediction of PCOS

The power of serum miRNA -320 expression levels to diagnose PCOS among studied subjects was evaluated using ROC analysis. The AUC was 0.861 (95% CI = 0.788–0.935) with sensitivity = 80%, specificity = 97.5%, and the cutoff values (0.7429), *P* < 0.001*, (Fig. 3). The positive predictive value of serum miRNA -320 for diagnosis of PCOS was 35% and the negative predictive value was 80%

#### The accuracy of serum ET-1for diagnosis of PCOS by ROC analysis

Regarding serum ET-1. The AUC was 0.882 (95% CI = 0.804–0.959) with sensitivity = 91.7%, specificity = 99.3%, and the cutoff values (5.66), *P* < 0.001*, (

**Fig. 4:**
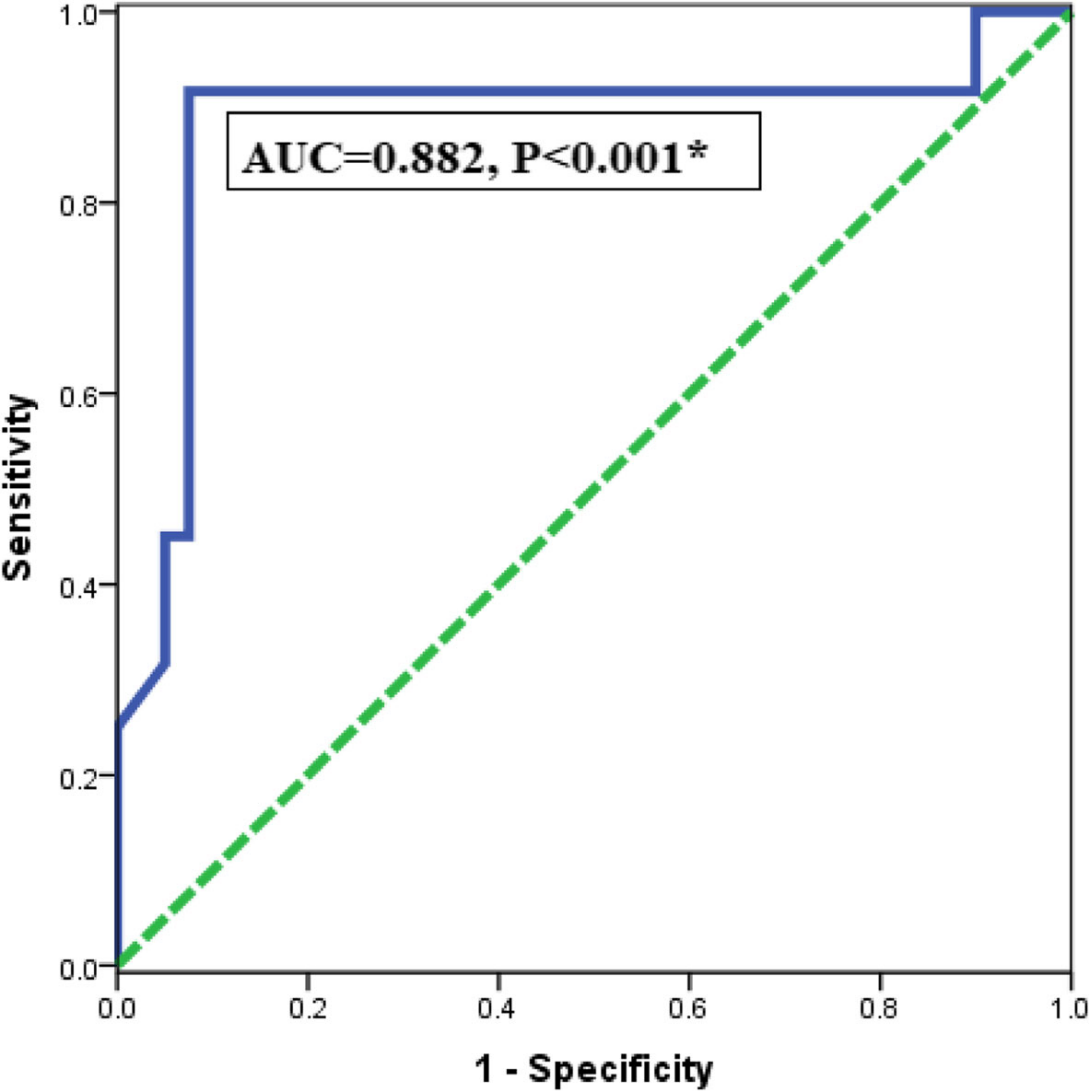
Receiver operating characteristic (ROC) curve for serum ET-1 level for prediction of PCOS

Fig. 4). The positive predictive value of serum ET-1for diagnosis of PCOS was 38.5% and the negative predictive value was 75%

## Discussion

Emerging evidence demonstrated that PCOS is a complex and heterogeneous endocrine condition characterized by hyperandrogenism and IR. Extensive data support a major role of IR as a common feature of PCOS, but not a diagnostic criterion of PCOS [12].

In polycystic ovaries of anovulatory women, the precise molecular mechanisms that regulate antral follicular development remain unclear, omics studies have indeed demonstrated that the growth of these follicles is typically arrested before a mature follicle would be expected to ovulate [13].

MicroRNAs are endogenous, small, non-coding, single-stranded, regulatory RNA molecules, composed of 20–24 nucleotides [14]. There are intriguing reports suggested that miRNAs had a key role in follicular development and steroidogenesis among PCOS women. Moreover, emerging data have demonstrated that serum miRNAs could serve as a non-invasive biomarker for PCOS, as they have been shown to be stable and resistant to nuclease activity and are easy to detect [15].

ET-1 gene is regulated by different levels of expression, and regulation at the post-transcriptional level by miRNAs in particular; miR-320 which negatively regulates expression of ET-1. The aim of the current study was to investigate the role of miRNA- 320 as a noninvasive biomarker of PCOS and to evaluate its possible relationship with IR as well as clinic-morphological features of PCOS.

The results of the current study showed statistically significant elevations of cardiometabolic risk factors; insulin resistance and dyslipidemia, as well as phenotype characteristic of PCOS, compared to controls. These results are in concordance with our previous studies [16–20].

The main finding of the present study is that the expression levels of miRNA − 320 in PCOS patients were significantly lower compared to the control group.

Our findings are in concordance with Long et al. they confirmed the downregulation of miR-320 in PCOS compared to controls [21].

We in this study attempted to pierce out the association between miRNA − 320 expression levels and IR in PCOS women, we detected that IR patients had significantly lower expression levels of miRNA − 320 compared to PCOS women without IR.

In agreement with our results, Wang et al. found decreased the levels of miRNA in the serum of diabetic individuals as well as in the cardiac microvascular cells of diabetic rodents [22].

Similar to our result, a study conducted by Feng and Chakrabarti found decreased the expression of microRNA 320 in hyperglycemia [23].

Conflicting data have been reported expression levels of miRNA − 320 in various cohorts of PCOS, diabetic and obese patients as well as in rodents.in the study conducted by Ling et al. revealed that miR-320 expression levels in insulin-resistant adipocytes were upregulated [24].

Sang et al. revealed that miR-320 is expressed at significantly lower levels in the follicular fluid of PCOS patients than in healthy controls [25]. Also, Yuan and Tan confirmed in their study the downregulation of microRNA-320 expression in the ovarian tissue in the PCOS–IR group compared to the control group. Interestingly, they supposed that microRNA-320 could inhibit IR in patients with PCOS through IRS-1 regulating the ERK1/2 signaling pathway [26].

The study conducted by Yin et al. suggested that miR-320 expression levels increased in granulosa cells and associated with follicle proliferation and steroidogenesis of the ovary during ovary development [27].

The controversy of results could be due to differences in sample and methodology. In our study, we aimed to explore the noninvasive diagnostic biomarkers miR-320 so, we measure the miR-320 expression level in serum but most of the published studies estimated the miR-320 expression level in ovarian tissue as well as follicular fluid.

Regarding serum levels of ET-1, PCOS patients had significantly higher levels compared to the control group. Diamanti-Kandarakis et al. found that women with PCOS had elevated ET-1 levels compared with the age-matched control group [28].

Among the PCOS group, IR patients had significantly higher serum levels of ET-1 compared to PCOS women without IR. In agreement with our results, previous studies detected a higher level of ET-1 in patients with IR for example, obesity [29], diabetes mellitus [30], as well as impaired glucose tolerance [31].

The results presented herein are innovative; as this study performs a robust evaluation of serum miRNA − 320 expression levels in regulating ET-1 level, we observed that there was a significantly negative correlation between serum miRNA − 320 expression levels and ET-1.

Our data are in line with previous findings reporting that miR-320 negatively regulates expression of ET-1 [23].

Emerging evidence shows that MicroRNAs can regulate gene expression in both normal and disease states by direct targeting of the messenger RNA and inhibition of its translation to a protein, and/or by interfering with the epigenome [32] miRNA-320 act through its target ET-1 and inhibit IR in patients with PCOS through IRS-1 regulating the ERK1/2 signaling pathway and control pathways involved in follicular maturation [33].
